# Peoria District Medical Society

**Published:** 1848

**Authors:** 


### ARTICLE II.

### PEORIA DISTRICT MEDICAL SOCIETY.

The annual meeting of this spirited association, was held in Peoria, on the 6th of June. There were about thirty members present.

The President Dr. F. A. McNeill, delivered the annual address, and Dr. Frye, a lecture on generation. Dr. Colburn made a report on the use of ether in obstetric practice. The officers for the ensuing year were elected, as follows:

*President*—Dr. E. M. Colburn, of Bloomington.

*Vice Presidents*—Drs. Thos. Hall, of Toulon, and Alanson Stockwell, of Tremont.

*Recording Secretary*—Dr. E. Andrew, of Peora.

*Corresponding Secretary*—Dr. J. Murray, of Peoria.

*Treasurer*—Dr. Edward Dickinson, of Peoria.

*Censors*—Drs. Sexton, of Galesburg, Arnold, of Peoria, Wilson, of Metamora, Quigly, of Perkin, and Christy, of Farmington.

*Delegates to the American Medical Association*, for the meeting on 2d Tuesday of May, 1849—Drs. Joseph C. Frye, Edward Dickinson, and Francis A. McNeill, of Peoria, and H. H. Sexton, of Galesburg.

The code of ethics of the American Medical Association, was adopted. Our friends of this society are fairly organized and under way, and we understand are exerting a most happy influence, in the extensive region of the country over which its members are scattered. From the subjects committed for investigation, we infer that they are at work right, for the advancement of science.

There are five standing committees, they are as follows:

1st. *On Medical Inquiry*—Drs. Murphy, Henry, Hall, McNeill, Wood, and Luce.

2d. *On Medical Statistics*—Drs. Hanneford, Sexton, Cooper, and Golliday.

3d. *On Indigenous Botany*—Drs. J. D. Arnold, Boal, Christy, Van Patten, Stockwell, and Rogers.

4th. *On Library and Museum*—Drs. E. Dickinson, Andrew, and McClure.

5th. *On Natural History, Mineralogy, and Geology*—Drs. N. S. Tucker, Wilson, and Hickman.

Two special committees were appointed.

1st. On the influence of malarious atmosphere, in the prevention and cure of pulmonary diseases—consisting of Dr. J. C. Frye.

2d. On the utility of anæsthetic agents—consisting of Drs. Andrew, Colburn, and Cook.

Six new members were admitted. [E.

